# Association Between Neutrophil-to-HDL Ratio and Full-Spectrum Dysglycemia: Insights from a Large Middle Eastern Population Study

**DOI:** 10.3390/healthcare13233021

**Published:** 2025-11-22

**Authors:** Abdulaziz M. Almuqrin, Abdulrahman F. Alrezaihi, Ali A. Aljasser, Ibrahim Alqarni, Yazeed Alshuweishi, Mohammad A. Alfhili

**Affiliations:** 1 Chair of Medical and Molecular Genetics Research, Department of Clinical Laboratory Sciences, College of Applied Medical Sciences, King Saud University, Riyadh 12372, Saudi Arabia; 2 Department of Clinical Laboratory Sciences, College of Applied Medical Sciences, King Saud University, Riyadh 12372, Saudi Arabia; 3 Department of Physiology, College of Medicine, King Saud University, Riyadh 11421, Saudi Arabia

**Keywords:** dysglycemia, neutrophil to high-density lipoprotein cholesterol ratio, general population, hyperglycemia, impaired fasting glucose

## Abstract

**Background:** Chronic low-grade inflammation and dyslipidemia contribute to metabolic disorders such as diabetes. Dysglycemia, an early stage of glucose dysregulation, reflects this interplay. The neutrophil-to-HDL cholesterol ratio (NHR) integrates inflammatory and lipid pathways, but its role in dysglycemia, particularly in Middle Eastern populations, remains unclear. This study evaluated the association between NHR and glycemic abnormalities, including impaired fasting glucose (IFG) and hyperglycemia (HG). **Methods:** A retrospective cross-sectional investigation involving 13,121 individuals at a major health setting in Saudi Arabia was conducted. The association between NHR and glycemic status was comprehensively evaluated, with subgroup analyses stratified by age and gender. Risk and diagnostic performance of NHR for dysglycemia were evaluated. Correlation and linear regression analyses were conducted to examine the relationships between NHR and fasting glucose, as well as HbA1c. **Results:** NHR levels were significantly associated with dysglycemia, showing progressive elevation from normoglycemia (5.30: 3.453–7.755) to IFG (6.00: 3.904–8.663) and HG (7.177: 4.917–10.17). Elevated NHR was more prevalent among individuals with dysglycemia and was associated with an increased risk of IFG (OR = 1.48, 95% CI: 1.36–1.61, *p* < 0.0001) and HG (OR = 2.38, 95% CI: 2.13–2.65, *p* < 0.0001). In the FBG context, NHR showed better discrimination of HG from NG than CRP (AUC = 0.650 vs. 0.570; *p* < 0.0001). **Conclusions:** Elevated NHR is significantly associated with dysglycemia, particularly hyperglycemia. These findings suggest that NHR may serve as a supportive tool for identifying individuals at increased risk of dysglycemia, especially in populations with limited diagnostic resources. Future longitudinal studies are warranted to validate its predictive and prognostic value in clinical practice.

## 1. Introduction

Diabetes is a chronic medical condition characterized by abnormally elevated glucose levels in the bloodstream resulting from inadequate insulin production or resistance. Several risk factors are involved in the development of diabetes, including reversible factors such as bad diets, smoking, and obesity, and irreversible factors, e.g., race, genetic predisposition, and age [1]. Diabetes is conventionally linked to various macrovascular and microvascular complications, e.g., coronary artery disease, myocardial infarction, retinopathy, stroke, and renal diseases [2]. Dysglycemia, encompassing IFG and HG, represents an early and clinically significant stage within this spectrum, which can progress to diabetes [3].

Diabetes is one of the top ten leading causes of death globally, accounting for health expenditures exceeding 727 billion dollars in 2017 [4]. It affects approximately 500 million individuals worldwide, and is expected to rise to 700 million within the next two decades [5]. Diabetes is a significant health concern in the Kingdom of Saudi Arabia, where epidemiological studies have estimated its prevalence among the Saudi population to be approximately 18%, which puts Saudi Arabia among the countries with the highest prevalence rates [6,7,8]. It has been estimated that healthcare expenditures of the diagnosed and undiagnosed Saudi diabetic population are approximately $7.2 billion [9].

One of the key factors in the development of diabetes is low-grade chronic inflammation. This condition is characterized by increased production of interleukins and chemokines, such as monocyte chemoattractant protein 1 (MCP-1), which activate and recruit monocytes and proinflammatory macrophages, leading to systemic inflammation [10]. Insulin resistance, a hallmark of diabetes, further exacerbates this process. The resulting oxidative injury, triggered by hyperglycemia and hyperlipidemia, initiates stress-signaling pathways that impair tissue sensitivity to insulin [11].

Several new-generation inflammatory indices derived from routine laboratory tests have recently been explored as inexpensive and accessible tools for predicting and prognosing various health disorders. Chronic low-grade inflammation and dyslipidemia are key features of dysglycemia [12]. Neutrophils are major mediators of inflammation and have been associated with insulin resistance, complications, and diabetes progression, whereas high-density lipoprotein cholesterol (HDL-C) exerts anti-inflammatory and antioxidant effects that help maintain glucose and lipid homeostasis [13,14]. Therefore, the neutrophil-to-HDL cholesterol ratio (NHR) has been recognized as a newly emerging index reflecting both inflammatory and lipid-related pathways. Recent studies have demonstrated the prognostic value of NHR in various health conditions, including cardiovascular mortality, hepatocellular carcinoma, and ischemic stroke [15,16,17].

To our knowledge, this is one of the first studies to comprehensively examine the association between NHR and the full spectrum of glycemic disturbances, including IFG and HG, particularly in Middle Eastern populations, which carry one of the highest global burdens of diabetes and related metabolic disorders. The present work integrates both fasting blood glucose (FBG) and HbA1c measurements to capture this full spectrum, providing a more comprehensive view of the short- and long-term dynamics linking inflammation, lipid metabolism, and glucose regulation. Many Middle Eastern nations face limitations in diagnostic resources, highlighting the need for simple and cost-effective biomarkers to facilitate early detection of dysglycemia. Accordingly, this large, population-based study conducted in Saudi Arabia addresses this need by evaluating NHR as a potential practical indicator of glycemic disturbances in the general population.

## 2. Materials and Methods

### 2.1. Study Population and Design

**Figure 1:**
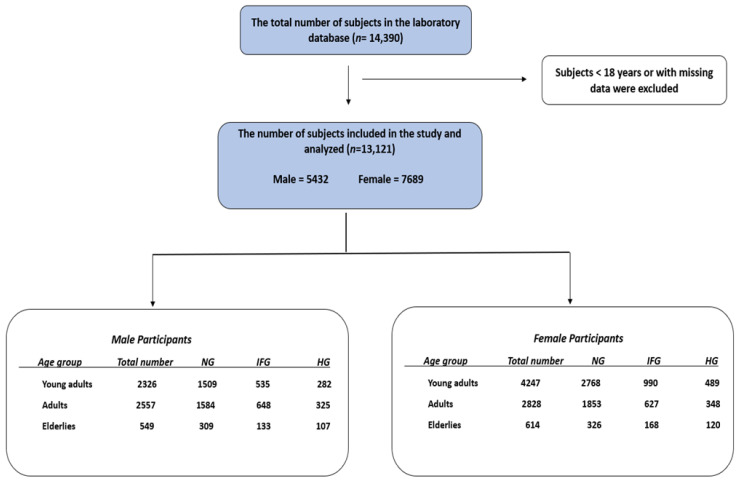
A schematic representation illustrating the study design.

Following ethical approval by the Biomedical Ethics Unit at Al Borg Diagnostics (protocol code #07/21; approval date: 27 December 2021), demographic and laboratory data from 14,390 adults representing the general Saudi population were obtained from a major diagnostic laboratory network that provides nationwide routine health testing services. Subjects younger than 18 or with missing data were excluded. Complete blood count (CBC) parameters were analyzed using the Sysmex XN-Series automated hematology analyzer (Sysmex Corporation, Kobe, Japan) to obtain CBC values, while FBG and HbA1c were measured using the ARCHITECT i1000SR analyzer (Abbott Laboratories, Abbott Park, IL, USA). All assays were conducted under routine laboratory conditions within the accredited quality-assurance framework of Al Borg Diagnostics. As shown in Figure 1, participants were categorized by age into three groups: young adults (18–39 years), adults (40–64 years), and the elderly (≥65 years). In accordance with ADA recommendations, normoglycemia (NG) was defined as FBG below 100 mg/dL, IFG as an FBG level between 100 and 125 mg/d, and HG as an FBG level of 126 mg/dL or above [18]. NHR was manually calculated as $Neutrophils(10^{3}cells/\mu L)÷HDL-C(mg/dL)$ [15]. NHR values > 5.830 were considered high (H-NHR), while values ≤ 5.830 were considered normal (N-NHR). Furthermore, participants with available HbA1c values were stratified into three categories based on their HbA1c levels: normoglycemic (<5.7%, HbA1c-NG), prediabetic (5.7–6.4%, HbA1c-PreDM), and diabetic (≥6.5%, HbA1c-DM) ranges [19].

### 2.2. Statistics

Because the extracted data were not normally distributed, nonparametric tests were utilized to perform the statistical analysis. Comparisons between two groups were performed using the Mann–Whitney U test, while comparisons among three or more groups were assessed using the Kruskal–Wallis test. The results were presented as medians with interquartile ranges (IQRs). Simple linear regression analysis was performed to evaluate the correlation between NHR and FBG or HbA1c values. MedCalc Statistical Software version 20.218 (MedCalc Software Ltd., Ostend, Belgium) was used to perform the risk assessment analyses, including the calculation of odds and prevalence ratios. The diagnostic performance of NHR for predicting glycemic abnormalities was assessed using receiver operating characteristic (ROC) analysis, with sensitivity, specificity, and area under the curve (AUC) values reported. The statistical analysis was performed using GraphPad Prism v9.2.0 (GraphPad Software, Inc., 10.0.1, San Diego, CA, USA). *p*-values < 0.05 were deemed statistically significant.

## 3. Results

### 3.1. Baseline Characteristics of the Study Population

In total, 13,121 subjects were involved in the presented study. The study population was categorized based on their glycemic status into 3 groups: NG, IFG, and HG. Of the total study population, males represented 25.9% in the NG group, 10.0% in the IFG group, and 5.4% in the HG group, while females comprised 37.7%, 13.6%, and 7.3%, respectively (Table 1). Age showed a significant progressive increase across the glycemic categories. Other routinely measured laboratory parameters are shown in Table 1.

### 3.2. NHR Levels Are Elevated in Dysglycemia and Associated with Higher FBG

**Figure 2:**
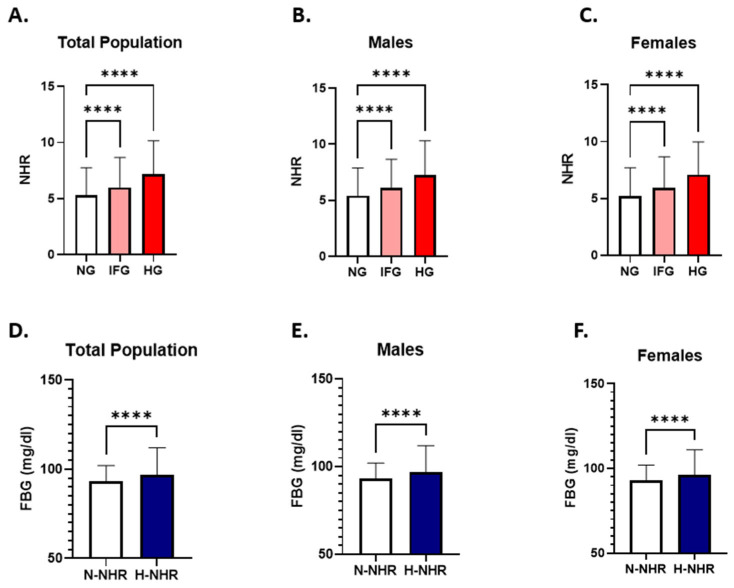
Evaluation of NHR patterns in relation to FBG concentrations. A comparison of NHR levels between NG, IFG, and HG groups is presented in the total study population (**A**), in males (**B**), and in female subjects (**C**). The difference in FBG concentrations between N-NHR and H-NHR groups in the total population (**D**), male subjects (**E**), and females (**F**) are shown. Asterisks mark the significance level; **** for *p* < 0.0001.

To assess the levels of NHR in light of glycemic status, NHR levels were compared across the NG, IFG, and HG groups. The analysis showed a significant increase in NHR levels in the IFG (6.00: 3.904–8.663) and HG groups (7.177: 4.917–10.17) compared to the NG subjects (5.30: 3.453–7.755) in the total population (Figure 2A).

A similar trend was observed after stratification by gender. Among male subjects, NHR were significantly elevated in IFG (6.137: 4.082–8.673) and HG (7.28: 4.979–10.32) groups compared to their NG counterparts (5.417: 3.591–7.876) (Figure 2B). Similarly, NHR levels showed a significant increase in IFG (5.927: 3.753–8.663) and HG (7.105: 4.822–9.986) female subjects in comparison to females in the NG group (5.224: 3.359–7.70) (Figure 2C).

To further evaluate the link between NHR and dysglycemia, FBG concentrations were analyzed in light of NHR levels in the study subjects. The analysis revealed FBG levels were significantly higher in the H-NHR group (97:88–112) compared to the N-NHR group (93: 87–102) (Figure 2D). This was true when males and females were analyzed separately (Figure 2E,F).

### 3.3. Age- and Gender-Stratified Analysis of NHR in Dysglycemia

**Figure 3:**
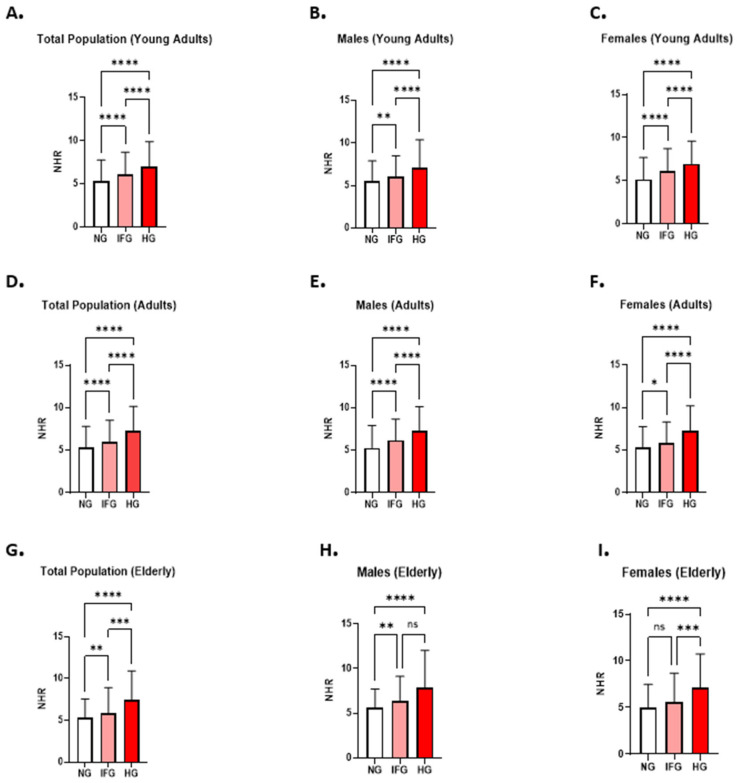
The combined effect of age and gender on NHR measures in relation to FBG. A comparison of NHR patterns between NG, IFG, and HG groups in young adults is illustrated for the total population (**A**), male subjects (**B**), and females (**C**). The changes in NHR levels between NG, IFG, and HG groups are shown for adults in the total population (**D**), in males (**E**), and in female participants (**F**). The difference in NHR patterns between NG, IFG, and HG groups in elderly subjects is presented for both genders (**G**), for males (**H**), and for females (**I**). Asterisks denote statistical significance levels: *p* < 0.05 (*), *p* < 0.01 (**), *p* < 0.001 (***), and *p* < 0.0001 (****); “ns” denotes non-significant difference.

Participants were then stratified based on their age and gender to evaluate the combined impact of age and gender on the NHR patterns. Among young adults, NHR levels were significantly elevated in the IFG (6.077: 4.00–8.684) and HG (6.974: 4.827–9.906) groups compared to the NG group (5.047: 3.244–7.274) (Figure 3A). Similar trends were observed when males and females were analyzed separately (Figure 3B,C).

Among adults, NHR levels were also significantly higher in the IFG (5.957: 3.781–8.528) and HG (7.306: 4.891–10.17) groups than in the NG group (5.277: 3.410–7.755) (Figure 3D). This pattern persisted across both genders, with comparable increases observed in both males and females (Figure 3E,F).

Among elderly participants, NHR levels were significantly elevated in the IFG (5.850: 3.923–8.937) and HG (7.459: 5.114–10.93) groups compared to the NG group (5.326: 3.480–7.556) (Figure 3G). Consistent results were obtained when both genders were considered separately (Figure 3H,I).

### 3.4. Correlation of NHR with FBG Concentration in the Study Population

**Figure 4:**
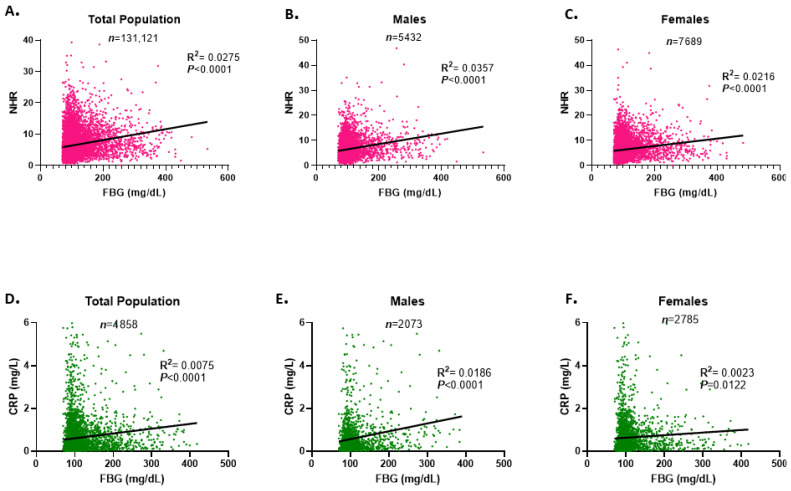
Correlation of NHR and CRP with FBG concentrations in the study population. Simple linear regression of the association between NHR and FBG concentrations is presented for both genders (**A**), males (**B**), and females (**C**). Simple linear regression of the association between CRP and FBG concentrations is presented for the total population (**D**), male subjects (**E**), and females (**F**). *n* represents the number of subjects included in each analysis.

Next, a comparative analysis of the correlations of NHR and the classical inflammatory marker, C-reactive protein (CRP), with FBG concentrations was performed. As shown in Figure 4A, linear regression analysis revealed a weak but statistically significant correlation between NHR levels and FBG concentrations in the total study population (R^2^ = 0.0275, *p* < 0.0001). Gender-specific analyses revealed a similar pattern, with a slightly stronger correlation in males than in females (R^2^ = 0.0357 vs. R^2^ = 0.0216) (Figure 4B,C). In contrast, CRP demonstrated a weaker association with FBG concentrations compared to NHR in both the overall population (R^2^ = 0.0075 vs. 0.0275) (Figure 4D) and in gender-stratified analyses (Figure 4E,F). These findings suggest that NHR could be a more sensitive indicator of dysglycemia-related inflammation than CRP.

### 3.5. Diagnostic Performance of NHR for Dysglycemia in Light of FBG Concentrations

**Figure 5:**
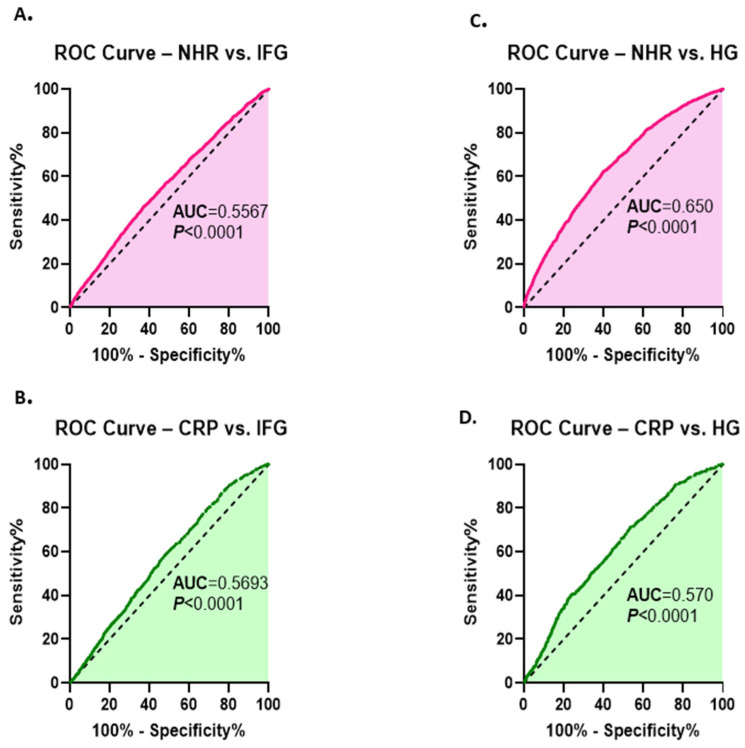
Diagnostic performance of NHR and CRP in discriminating dysglycemia based on FBG. ROC curves of NHR (**A**) and CRP (**B**) for differentiating subjects with IFG from the NG group are presented. ROC curves of NHR and CRP for differentiating HG subjects from NG individuals are illustrated in (**C**) and (**D**), respectively.

A comparative ROC curve analysis was conducted to evaluate the diagnostic performance of NHR and CRP in differentiating dysglycemic individuals from NG participants. Both markers showed limited discriminatory ability for IFG (Figure 5A,B). However, NHR demonstrated superior performance in differentiating HG from NG (AUC = 0.650, *p* < 0.0001) compared with CRP (AUC = 0.570, *p* < 0.0001) (Figure 5C,D).

### 3.6. Evaluating NHR Patterns Across HbA1c Ranges

To further assess the relationship between NHR and dysglycemia, participants were stratified into three groups based on their HbA1c levels: HbA1c-NG (<5.7%), HbA1c-PreDM (5.7–6.4%), and HbA1c-DM (≥6.5%).

**Figure 6:**
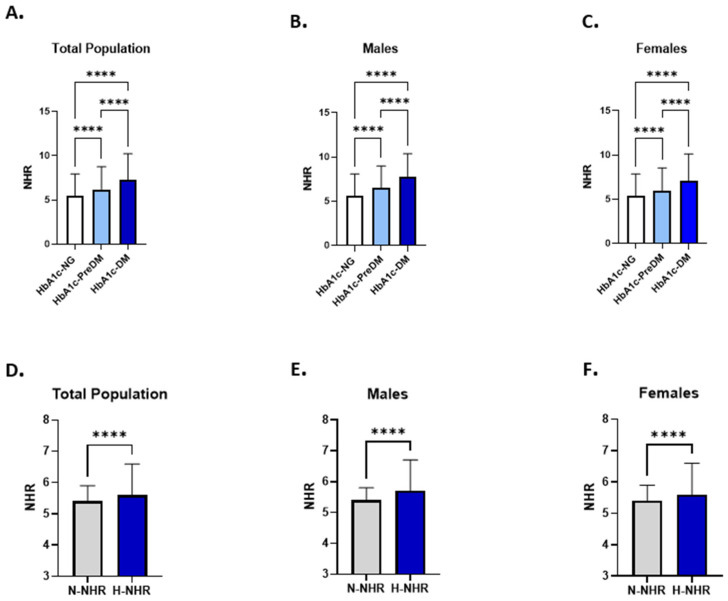
Evaluation of NHR patterns in relation to HbA1c levels. A comparison of NHR levels across the HbA1c-NG, HbA1c-PreDM, and HbA1c-DM groups is shown for the total study population (**A**), for males (**B**), and female participants (**C**). A comparison of HbA1c measures between the N-NHR and H-NHR groups in the total population (**D**), in male subjects (**E**), and in females (**F**). Asterisks mark the significance level; **** for *p* < 0.0001.

NHR levels were significantly higher in the HbA1c-PreDM (6.192: 4.167–8.757) and the HbA1c-DM groups (7.297: 5.053–10.22) compared with the HbA1c-NG group (5.472: 3.51–7.949) (Figure 6A). Similar patterns were observed when males and females were analyzed separately (Figure 6B,C). To further assess the relationship between NHR and dysglycemia, HbA1c levels were analyzed in relation to NHR levels. HbA1c values were significantly higher in the H-NHR group (5.60: 5.20–6.675) compared with the N-NHR group (5.40: 5.10–5.90) (Figure 6D). Similar trends were observed when males and females were analyzed separately (Figure 6E,F).

### 3.7. NHR Shows Stronger Correlation with HbA1c than CRP

**Figure 7:**
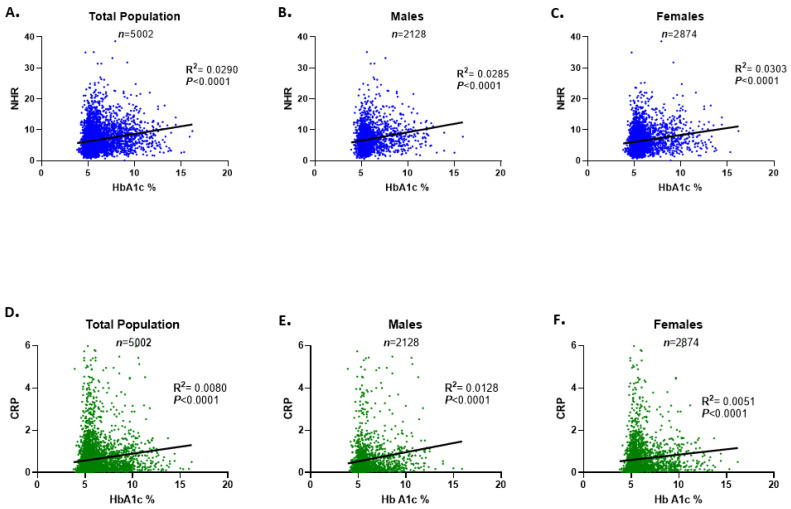
Correlation of NHR and CRP with HbA1c in the study population. Simple linear regression of the relationship between NHR and HbA1c is presented for both genders (**A**), male subjects (**B**), and females (**C**). The correlation of NHR with HbA1c is presented for the total population (**D**), males (**E**), and females (**F**). *n* represents the number of subjects included in each analysis.

Although simple linear regression revealed a weak correlation between NHR and HbA1c (R^2^ = 0.029) and between CRP and HbA1c (R^2^ = 0.008) in the total population, the association with HbA1c was comparatively stronger for NHR than for CRP (Figure 7A,D). Similar trends were observed in gender-specific analyses, where NHR consistently showed better correlations with HbA1c than CRP in both males and females.

### 3.8. Diagnostic Performance of NHR for Dysglycemia in Relation to HbA1c

**Figure 8:**
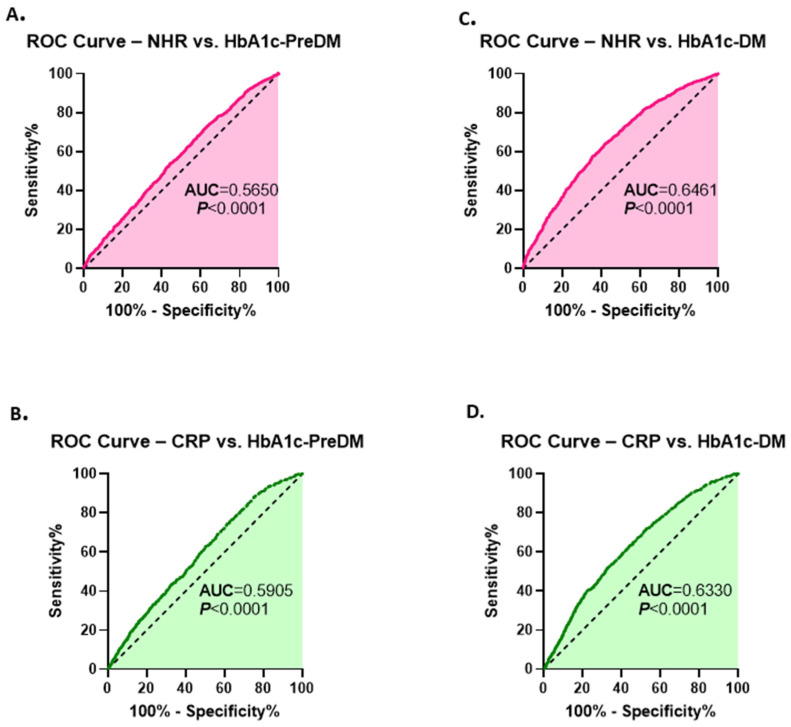
Diagnostic performance of NHR and CRP in discriminating dysglycemia based on HbA1c. ROC curves of NHR (**A**) and CRP (**B**) for distinguishing HbA1c-PreDM from HbA1c-NG individuals are shown. ROC curves of NHR and CRP for differentiating HbA1c-DM subjects from HbA1c-NG participants are illustrated in (**C**) and (**D**), respectively.

As shown in Figure 8A,B, NHR and CRP exhibited poor and comparable diagnostic ability in identifying individuals with HbA1c-PreDM, consistent with their performance using FBG (Figure 5A,B). However, both markers demonstrated similar better performance for predicting subjects with HbA1c-DM (Figure 8C,D). These findings suggest that while CRP likely reflects longer-term glycemic burden, NHR may capture both short- and longer-term metabolic inflammatory changes, as it was associated with hyperglycemia defined by both FBG and HbA1c.

### 3.9. Elevated NHR Levels Are More Prevalent Among Dysglycemic Subjects

As demonstrated in Table 2, normal NHR measures were more common among the NG participants compared to the IFG and HG groups. In contrast, the prevalence of elevated NHR increased progressively across the glycemic spectrum, rising from 43.6% in the NG group to 52.0% in subjects with IFG and 64.8% in the HG group. A comparable trend was observed in the sex-stratified analysis, with elevated NHR measures being consistently increased in both males and females across the glycemic spectrum.

### 3.10. Risk Assessment Analysis of the Association Between NHR and Dysglycemia Forms

As shown in Table 3, the risk assessment analysis revealed that elevated NHR levels were significantly associated with an increased risk of IFG in the total population (PR = 1.22, 95% CI: 1.17–1.27, *p* < 0.0001), in males (PR = 1.18, 95% CI: 1.10–1.25, *p* < 0.0001), and females (PR = 1.20, 95% CI: 1.13–1.26, *p* < 0.0001). Similarly, the chance of having IFG was 1.48 times higher among subjects with elevated NHR in both genders, 1.39 times in males, and 1.41 times in females.

In addition, the analysis showed that increased NHR levels were linked with a higher risk of HG in both genders (PR = 1.48, 95% 1.42–1.55, *p* < 0.0001), in males (PR = 1.46, 95% 1.37–1.56, *p* < 0.0001), and in female subjects (PR = 1.49, 95% 1.41–1.58, *p* < 0.0001). The likelihood of subjects with elevated NHR falling into the HG group was 2.38 (95% CI: 2.13–2.65, *p* < 0.001). Gender-specific analysis showed male and female participants with increased NHR levels had 2.68 (95% CI: 2.25–3.19, *p* < 0.001) and 2.37 (95% CI: 2.05–2.73, *p* < 0.001) times the chance of falling in the HG group, respectively.

### 3.11. Regression Analysis of the Association Between NHR and Glycemic Markers

As shown in Table 4, NHR demonstrated significant positive associations with both FBG and HbA1c in unadjusted and age- and gender-adjusted regression models. For FBG, the association remained consistent after adjustment (unadjusted estimate: 0.01745, 95% CI: 0.01567–0.01922; adjusted estimate: 0.01733, 95% CI: 0.01555–0.01910; both *p* < 0.0001). A similar pattern was observed for HbA1c (unadjusted estimate: 0.4871, 95% CI: 0.4090–0.5653; adjusted estimate: 0.4810, 95% CI: 0.4025–0.5595; both *p* < 0.0001). These findings highlight a significant and independent association between NHR and glycemic markers, supporting its potential as an indicator of dysglycemia.

## 4. Discussion

The neutrophil-to-HDL-cholesterol ratio (NHR) has emerged as a potential inflammatory marker that can be easily calculated from routine laboratory parameters and has been investigated as a prognostic tool in various health disorders [15,20,21,22]. However, few studies have assessed the association between NHR and dysglycemia in general population settings, and research investigating its relationship across the full spectrum of dysglycemia remains particularly scarce. This gap is especially relevant to Middle Eastern populations, which face one of the highest global burdens of diabetes yet often have limited diagnostic resources due to regional instability and conflicts, highlighting the importance of evaluating simple and accessible markers such as NHR for early detection of glycemic disturbances.

The present study demonstrated a significant elevation of NHR levels in both IFG and HG groups compared with NG participants, with the increase being more pronounced in the HG group across both glycemic markers. Elevated NHR measures were also more prevalent among individuals with dysglycemia, and risk assessment analyses revealed that higher NHR levels were associated with an increased risk of both IFG and HG, with stronger associations observed for hyperglycemia. Furthermore, regression analyses confirmed significant positive relationships between NHR and both FBG and HbA1c after adjustment for age and sex, supporting the independence of this association.

ROC curve analysis showed that NHR and CRP had comparable performance when assessed using HbA1c, whereas NHR demonstrated superior ability to distinguish hyperglycemia in the FBG context, suggesting that NHR may capture both short and long-term metabolic-inflammatory changes.

The results of the current study are consistent with previous reports demonstrating the clinical relevance of NHR in metabolic and comorbid conditions. A large cohort study by Lin-Liu et al. showed that elevated NHR levels were associated with increased risk and poorer prognosis of cardiovascular disease among subjects with IFG in the Chinese population [23]. An investigation, including 991 subjects, showed that NHR levels were significantly higher in patients with active pulmonary tuberculosis and type 2 diabetes mellitus (T2DM) compared to those without diabetes, suggesting that elevated NHR is an independent risk factor for active pulmonary tuberculosis in patients with T2DM [24]. Another report by Ren et al. demonstrated a positive correlation between NHR and biochemical indicators of myocardial ischemia, such as cTnI (cardiac-specific troponin I), CK-MB (creatine kinase–myocardial band), and LDH (lactate dehydrogenase), in patients with acute coronary syndrome and T2DM. The study suggested NHR could independently predict the risk of acute coronary syndrome in individuals with T2DM [25]. In a large U.S. population-based study, Tao et al. reported that elevated NHR was associated with a higher prevalence of diabetes as well as increased all-cause and cardiovascular mortality among diabetic patients [26]. Furthermore, recent studies conducted in Chinese populations have reported a positive association between NHR and the incidence of diabetes, as well as metabolic syndrome components, including FBG abnormalities [27,28]. The current study adds to this evidence by evaluating NHR across the full spectrum of dysglycemia using both fasting glucose and HbA1c classifications in a Middle Eastern population for the first time.

Low-grade inflammation is increasingly recognized as a central factor in the development of metabolic disorders and organ dysfunction in the context of chronic hyperglycemia. The link between inflammation and diabetes is complex, with inflammatory processes contributing both to disease progression and to the onset of complications [10]. Elevated leukocyte counts serve as a reliable marker of subclinical inflammation and have been associated with the development of IFG and T2DM. Several studies have demonstrated a positive correlation between increased leukocyte count and the development of IFG and T2DM [29,30,31,32].

Neutrophils are the major subset of white blood cells and are strongly implicated in chronic inflammatory conditions, including cardiovascular disease, insulin resistance, and T2DM [30,33,34]. In a cross-sectional cohort study, Zhang et al. found a positive association between elevated neutrophil count and the incidence and progression of chronic kidney disease in patients with diabetes. Similarly, a study of 30,793 Korean participants found a significant association between higher neutrophil counts and both the presence and severity of diabetic retinopathy, suggesting that subclinical neutrophil-mediated inflammation contributes to microvascular complications of diabetes [35]. In addition, several reports have demonstrated a positive correlation between an increased neutrophil count and adverse CVD outcomes, particularly in individuals with T2DM, suggesting that monitoring neutrophil count may be useful in managing the progression of CVD in T2DM patients [36,37,38].

Neutrophils are the most abundant leukocytes, yet the causal link between their elevation and obesity-related insulin resistance or T2DM is not fully understood, although several mechanisms have been proposed [39,40]. In an in vivo study, Hanses et al. reported a significant increase in neutrophil counts associated with reduced apoptosis in diabetic mice, suggesting that hyperglycemia may prolong inflammation the diabetic mice through excessive production of pro-inflammatory cytokines by neutrophils [41]. Neutrophils can further exacerbate chronic inflammation via the release of elastase, which amplifies inflammatory responses. Talukdar et al.showed that neutrophil elastase causes cellular insulin resistance in mice fed a high-fat diet, and eliminating neutrophil elastase led to less tissue inflammation, improved glucose tolerance, and increased insulin sensitivity [42]. The effects of hyperglycemia on neutrophils are complex, involving alterations in cellular metabolism such as the shunting of glucose into different pathways, leading to modifications in transcription factors and cytokine production, and continuous feed-forward of exacerbated chronic inflammation in diabetes [43,44,45].

Dyslipidemia is frequently observed with chronic hyperglycemia, and several studies have linked low levels of HDL cholesterol (HDL-C) with an increased risk of T2DM [46,47,48]. In a large population-based study, Lui DTW et al. demonstrated that low HDL-C levels were significantly associated with an increased risk of major adverse cardiovascular events and mortality among individuals with T2D [49]. Furthermore, several studies revealed a positive correlation between dyslipidemia, involving low HDL-C, and the development of diabetic nephropathy in T2DM patients [50,51,52,53]. Indeed, HDL-C is involved in regulating plasma glucose levels by enhancing the release of insulin from pancreatic β-cells and modulating the absorption of glucose in skeletal muscle [54,55], underscoring its critical role in the pathophysiology of dysglycemia, from early impaired glucose regulation to overt T2DM.

Chronic hyperglycemia is associated with the generation of inflammatory mediators and oxidative stress, partly through its positive correlation with the oxidation of LDL particles [56]. Oxidized LDL promotes inflammation by enhancing leukocyte recruitment, stimulating the release of pro-inflammatory cytokines, and increasing the production of reactive oxygen species (ROS) [57]. HDL particles exhibit anti-inflammatory roles through their ability to protect LDL particles from oxidation by removing and inactivating oxidized lipids from LDLs and stimulating the production of Nitric oxide (NO), which serves as an antioxidant [57,58]. In addition, HDL particles contain various lipid and protein components with antioxidative properties, including apoA-I, apoE, and apoM, as demonstrated by animal studies [59,60,61]. Therefore, low HDL-C levels reduce these protective effects, contributing to enhanced oxidative stress and inflammation in chronic hyperglycemia.

The present study has several strengths, including its large sample size and the automated acquisition of laboratory results, which enhance data accuracy. Another advantage is the homogeneity of the study population, which minimizes potential confounding due to ethnic variability. Furthermore, by utilizing data from the general population rather than from clinical patients, our findings are less prone to selection bias and more generalizable to broader public health settings. Nevertheless, this study has multiple limitations. Given the cross-sectional design, causal inferences cannot be established. In addition, some important anthropometric and clinical variables, such as BMI and comorbidities, were not available. Other relevant information, including lifestyle behaviors and medication use, was also lacking, which may have introduced residual confounding. Future longitudinal studies with more comprehensive clinical data and different populations are needed to confirm and expand upon these findings.

## 5. Conclusions

In conclusion, this large general population-based study demonstrated a significant association between elevated NHR and dysglycemia, with the strongest associations observed in hyperglycemia. By integrating both glycemic markers in the analysis, the study captures the full spectrum of glycemic disturbance, including early alterations such as IFG. In FBG-based assessments, NHR exhibited better discriminatory ability for hyperglycemia than the classical inflammatory marker CRP. To our knowledge, this is the first study to evaluate NHR in this context within a Middle Eastern population, a region with one of the highest global burdens of diabetes, and where access to advanced diagnostic resources may be limited. Given its simplicity, NHR may represent a practical adjunctive marker for identifying individuals at increased risk of dysglycemia. Future longitudinal studies are needed to establish its prognostic role and clinical utility.

## Figures and Tables

**Table 1:** Baseline characteristics of the study population.

Characteristic	NG Group	IFG Group	HG Group	*p*-Value
Demographics				
Age (years)	39 (33–50)	40 (34–52)	41 (34–57)	<0.0001
Males, *n* (%)	3402 (25.93%)	1316 (10.03%)	714 (5.44%)	-
Female, *n* (%)	4947 (37.70%)	1785 (13.60%)	957 (7.29%)	-
Lab parameters				
Free T4 (ng/dL)	1 (0.92–1.09)	0.99 (0.91–1.08)	1 (0.92–1.11)	<0.0001
TSH (mIU/L)	1.76 (1.17–2.70)	1.82 (1.19–2.79)	1.85 (1.18–2.78)	0.0611
ALT (U/L)	17 (13–26)	22 (15–31)	22 (16–32)	<0.0001
AST (U/L)	18 (15–23)	19 (16–24)	18 (15–24)	<0.0001
Hemoglobin (g/dL)	14.10 (12.9–15.50)	14.70 (13.30–15.90)	14.80 (13.50–15.90)	<0.0001
Chloride (mEq/L)	105 (103–107)	105 (102–107)	102 (100–105)	<0.0001
Calcium (mg/dL)	9.60 (9.30–9.90)	9.60 (9.30–9.90)	9.60 (9.30–9.90)	0.09
Total Bilirubin (mg/dL)	0.56 (0.40–0.78)	0.54 (0.93–0.74)	0.5 (0.38–0.69)	<0.0001

Abbreviations: NG, normoglycemic group; IFG, impaired fasting glucose group; HG, hyperglycemic group; Free T4, free thyroxine; TSH, thyroid-stimulating hormone; ALT, alanine aminotransferase; AST, aspartate aminotransferase.

**Table 2:** Prevalence of Normal and Elevated NHR Measures According to Glycemic Status in the Study Population.

Parameter	NG	IFG	HG
Both genders			
N-NHR	56.44%	45.44%	35.25%
H-NHR	43.56%	51.98%	64.75%
Males			
N-NHR	54.85%	46.66%	33.75%
H-NHR	45.15%	53.34%	66.25%
Females			
N-NHR	57.53%	49.02%	36.36%
H-NHR	42.47%	50.98%	63.64%

Abbreviations: NG, normoglycemic group; IFG, impaired fasting glucose group; HG, hyperglycemic group; N-NHR, normal neutrophil-to-HDL cholesterol ratio; H-NHR, high neutrophil-to-HDL cholesterol ratio.

**Table 3:** Elevated NHR Levels Are Associated With a Higher Risk of Dysglycemia.

		Score	95% CI	Z Statistic	Significance Level
	PR				
	Both genders	1.2249	1.1753–1.2766	9.622	*p* < 0.0001
	Males	1.1815	1.1097–1.2579	5.217	*p* < 0.0001
	Females	1.2004	1.1352–1.2694	6.407	*p* < 0.0001
IFG	OR				
	Both genders	1.4822	1.3634–1.6114	9.232	*p* < 0.0001
	Males	1.389	1.2225–1.5780	5.046	*p* < 0.0001
	Females	1.4088	1.2638–1.5704	6.186	*p* < 0.0001
	PR				
	Both genders	1.4864	1.4239–1.5517	18.074	*p* < 0.0001
	Males	1.4673	1.3761–1.5644	11.717	*p* < 0.0001
	Females	1.4984	1.4142–1.5876	13.703	*p* < 0.0001
HG	OR				
	Both genders	2.38	2.1336–2.6548	15.55	*p* < 0.0001
	Males	2.6851	2.2540–3.1987	11.061	*p* < 0.0001
	Females	2.3705	2.0541–2.7357	11.808	*p* < 0.0001

**Table 4:** Regression Analysis of NHR in Relation to FBG and HbA1c.

Marker	Model	Estimate	95% CI	*p*-Value
FBG (mg/dL)	Unadjusted	0.01745	0.01567–0.01922	<0.0001
	Adjusted	0.01733	0.01555–0.01910	<0.0001
HbA1c %	Unadjusted	0.4871	0.4090 to 0.5653	<0.0001
	Adjusted	0.481	0.4025 to 0.5595	<0.0001

## Data Availability

Data are available from the corresponding author upon reasonable request, and with permission of Al Borg Diagnostics.
